# Trauma in Children during Lockdown for SARS-CoV-2 Pandemic. A Brief Report

**DOI:** 10.3390/children8121131

**Published:** 2021-12-04

**Authors:** Daniela Dibello, Marcella Salvemini, Carlo Amati, Antonio Colella, Giusi Graziano, Giovanni Vicenti, Biagio Moretti, Federica Pederiva

**Affiliations:** 1 Unit of Pediatric Orthopaedics and Traumatology Giovanni XXIII Children’s Hospital, University of Bari, 70126 Bari, Italy; giannicaizzi@libero.it (M.S.); dr.carloamati@gmail.com (C.A.); colella31@gmail.com (A.C.); 2 Orthopedic & Trauma Unit, Department of Basic Medical Sciences, Neuroscience and Sense Organs, School of Medicine, University of Bari Aldo Moro, 70124 Bari, Italy; giusi.graziano78@gmail.com (G.G.); dott.gvicenti@gmail.com (G.V.); biagio.moretti@uniba.it (B.M.); 3 Pediatric Surgery Department, “Vittore Buzzi” Children’s Hospital, 20154 Milano, Italy; federica.pederiva@asst-fbf-sacco.it

**Keywords:** trauma, children, lockdown, pandemic, SARS-Co-V-2

## Abstract

Purpose: The national lockdown established by the Italian government began on the 11th of March 2020 as a means to control the spread of SARS-CoV-2 infections. The purpose of this brief report is to evaluate the effect of the national lockdown on the occurrence and characteristics of trauma in children during lockdown. Methods: All children admitted to our paediatric orthopaedic unit with a diagnosis of fracture or trauma, including sprains and contusions, between 11 March 2020 and 11 April 2020, were retrospectively reviewed. Their demographic data, type of injury, anatomical location and need for hospitalisation were compared with the equivalent data of children admitted for trauma in the same period of 2018 and 2019. Results: Sixty-nine patients with trauma were admitted in 2020, with a significant decrease in comparison with 2019 (*n* = 261) and 2018 (*n* = 289) (*p* < 0.01). The patients were significantly younger, and the rate of fractures significantly increased in 2020 (*p* < 0.01). Conclusions: Home confinement decreased admissions to the emergency department for trauma by shutting down outdoor activities, schools and sports activities. However, the rate of fractures increased in comparison with minor trauma, involved younger children and had a worse prognosis.

## 1. Introduction

The vast majority of paediatric injuries in emergency rooms are fractures, which are common during childhood [1,2] and are mostly caused by trauma while practising sports or playing [2]. Such injuries have a great impact on the child’s and family’s daily life and carry significant social and economic consequences, both in the short- and long term [1].

In an attempt to counteract the fast spread of the infection caused by SARS-CoV-2, the Italian government declared a national lockdown on the 9 March 2020 with consequent closure of schools, gymnasiums and all commercial and industrial activities, except for those deemed essential [3,4].

This is a brief report outlining the effects of the national lockdown on the occurrence and characteristics of trauma seen in children, comparing first aid access in our hospital from 11 March to 11 April in 2018, 2019 and 2020 (the hardest restriction period). Data in the same period for 2016 and 2017 were actually under investigation. Our main hypothesis was that the lockdown drastically reduced the total number of fractures in children and that only major traumas were brought to our attention at our second-level paediatric trauma centre.

## 2. Methods

After approval was received from the Institutional Research Committee, all children who came to our paediatric orthopaedic unit with a diagnosis of fracture or trauma, including sprains and contusions, between 11 March and 11 April 2020, were retrospectively reviewed. Demographic data, type of injury, anatomical location and the need for hospitalisation were recorded. Patients with polytrauma or with associated neurological impairment were excluded. Children admitted for fracture or trauma in the same interval of time in 2018 and 2019 were used as controls. The diagnosis was confirmed in all cases by physical examination and plain radiograph, without the need to perform a CT scan or MRI. The results were expressed as percentages or as means ± SD, and both groups were compared by nonparametric Mann–Whitney, chi-squared or Fisher tests, as appropriate, with a threshold of significance at *p* < 0.05. The analyses were performed using R software (version 3.5.2).

## 3. Results

Sixty-nine children (30 female and 39 male) presented during the study period in 2020 with a trauma involving the upper limb in 68%(*n =* 47), the lower limb in 28% (*n* = 19) and the spine in 4% (*n* = 3) of the cases. In the correspondent period in 2019, 260 children (100 female and 160 male) were treated for trauma of the upper limb in 55% (*n* = 143) of the cases, the lower limb in 36% (*n* = 94) and the spine in 9% (*n* = 23). In 2018, 289 children (112 female and 177 male) were treated for trauma of the upper limb in 57% (*n* = 165) of the cases, the lower limb in 35% (*n* = 101) and the spine in 8% (*n* = 23). The mean age of the patients treated in the emergency department in 2020 (6.83 ± 4.06) was significantly lower when compared with 2019 (10.84 ± 4.23) and 2018 (9.95 ± 4.42; *p* < 0.001). In all cases, the number of patients admitted to our emergency room in 2020 was significantly lower than in 2019 and 2018 (*p* < 0.001).

**Figure 1:**
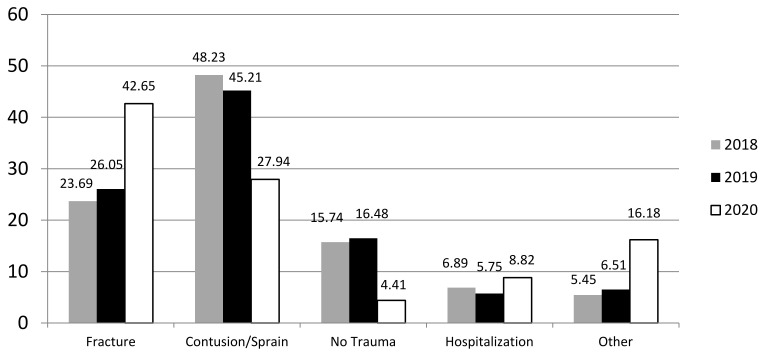
Comparison between 2018, 2019 and 2020 in terms of type of injury and need for hospitalization (in percentages).

**Figure 2:**
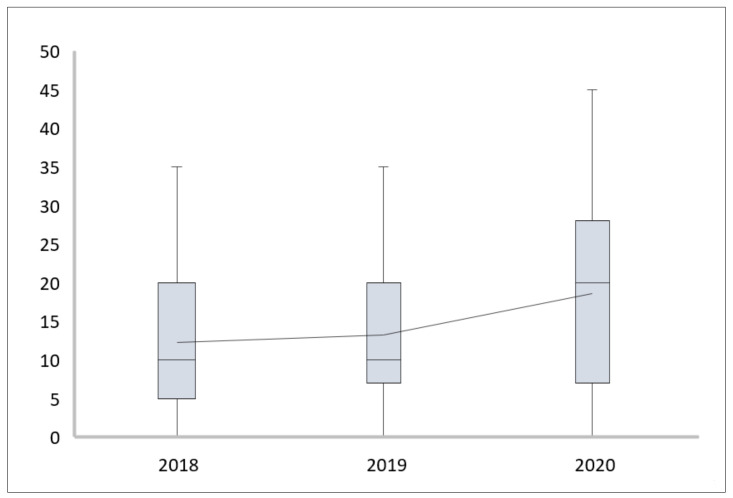
Comparison between 2018, 2019 and 2020 in terms of prognosis of the trauma.

In 2020, the number of fractures (29) was significantly higher in percentage in comparison with 2019 (67) and 2018 (68) when contusions and sprains were the most common lesions (*p* < 0.01) (Figure 1). On the other hand, although the need to admit the patient to the ward was similar across all years, the prognosis (as estimated by the orthopaedic surgeon who treated the patient in the emergency room) for the trauma was significantly worse in 2020 (*p* < 0.05) (Figure 2). While pain or functional diseases not related to trauma decreased in our paediatric orthopaedic unit, major trauma with complicated wounds, tendon involvement and other injuries that required a multidisciplinary evaluation increased (“other” in Figure 1). Nevertheless, the total number of trauma and injuries in 2020 decreased compared to 2019 and 2018.

**Figure 3:**
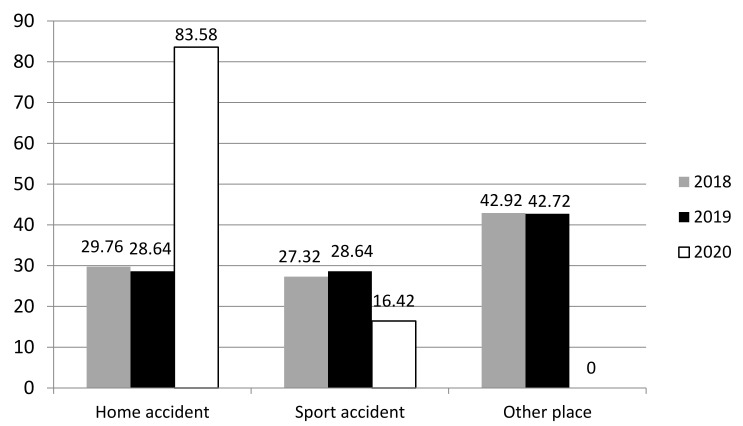
Comparison between 2018, 2019 and 2020 in terms of place in which trauma occurred (in percentages).

In 2020, the trauma occurred mostly at home (83.6% *n* = 58) and sport-related injuries were significantly lower (16.4% *n* = 11) in comparison with 2019 (28.7% *n* = 75) and 2018 (29.8% *n* = 87). In 2019 and 2018, the trauma more frequently happened away from home and was not sport-related (42.7% *n* = 111 and 42.9% *n* = 124, respectively) but due to play-related activities. Of the remaining cases for 2019 and 2018, half occurred at home (28.7% *n* = 75 and 29.7% *n* = 86, respectively) and the other half (28.6% *n* = 74 and 27.3% *n* = 79, respectively) were sport-related (Figure 3). However, no significant differences were found when comparing 2018 and 2019 in terms of the type of injury, prognosis and location of the trauma.

## 4. Discussion

Twelve percent of the admissions per year to the paediatric emergency department are due to musculoskeletal injuries [2,5]. The vast majority of these injuries are skeletal fractures, which cause significant morbidity to children and are an expensive public health issue. The overall rate of fractures is increasing despite the implementation of guidelines to prevent injuries and the campaign to raise parents’ awareness of the subject [2,6,7].

The Italian national lockdown from the 11 March 2020 meant children spent most of their time indoors and were not allowed to practice outdoor sports or activities. The first outcome of the stay-at-home order was a significant decrease in admissions (76% less in comparison with 2019, and 77% in comparison with 2018) to the emergency department for trauma. Moreover, trauma usually happening at school or practising sports almost disappeared during the lockdown. Most of the trauma in 2020 occurred at home, while in 2019 and 2018, they mostly happened outdoor while playing. This result highlights how significantly sports and play impact the incidence of fractures in children.

The patients treated in 2020 were significantly younger than the ones seen in 2019 and 2018. One explanation is that the lockdown eliminated all the trauma occurring during school time or related to sports. During this time, older children, now staying at home, spent more time playing videogames, watching TV or following lessons online, whereas preschool-aged children did not change their activities at home, such as running or jumping up and down from couches and beds. This could explain why the percentage of the age of trauma is inversed in 2020 in comparison to 2019 and 2018.

The rate of fractures increased in percentage in 2020 and the prognosis using days of hospitalisation worsened. This data could be explained by the fact that parents preferred to treat minor traumas at home without visiting a hospital for diagnosis and treatment. The distribution of the site of fractures was not modified by the lockdown, and upper limb fractures continued to be the most usual ones at both endpoints. Forearm fractures are the most common ones in children, accounting for 40–50% of all fractures during childhood [1,8,9,10]. The distal third of the forearm, including the radius and/or ulna, is involved in 75% of the cases [11,12] because of the increased body mass during their growth and development together with decreased bone mineral content [5,6,9,11].

Our results were consistent with the finding of other groups [13,14,15,16,17,18,19,20,21,22], although only a few of them analysed a paediatric population. Our study, in fact, demonstrates how a lockdown could differently affect children according to the age group.

These data lead to several considerations: as previously said, during the lockdown, a lot of minor traumas were likely treated at home without reaching a hospital or calling for medical care. In this scenario, a hypothesis is that many children were treated with only analgesics and rest at home. In the future, to limit the access to a second-level trauma care hub, children with minor trauma (e.g., the frequent torus fractures of the distal radius) could be treated in a first-level emergency spot where alternatives to a plaster cast treatment could be used (especially if an orthopaedic specialist is not available). In this way, we are conducting a study about the efficacy of treating children with torus distal radius fractures with an easy-to-use 3D-printed splint in place of the classic plaster cast usually moulded by an orthopaedic specialist and a dedicated nurse. Results collected to date showed faster treatment in the emergency room, improved childhood activities during recovery and high satisfaction for parents and children without any complication or delay in the healing process as seen for splints in previous studies [23,24]. As we can easily expect new epidemic waves in the near future, changing medical care modalities for minor trauma in children (at the moment, the least vaccinated population) could improve our attempts to limit the spread of the virus.

Another consideration that could be extrapolated from these data is related to our experience in orthopaedic fast-tracking: in our hospital, in fact, after the nursing triage in the emergency room, children with uncomplicated monosegmental trauma are sent directly to our unit. Next, an orthopaedic surgeon evaluates the case, visits the child, requires radiographs and eventually CT scans and then decides for immobilisation or hospitalisation. In the first case, the patient is discharged directly home. This consolidated pathway, already described in the literature [25], demonstrates a shorter stay in the hospital, virtually no waiting time in an emergency room (where otherwise healthy children could be exposed to those with fevers, coughs or colds) and higher satisfaction for parents and children. In these months of worldwide strategies for containment efficiency and spread control for the COVID-19 pandemic, in our opinion, every attempt to reduce the risk of contagion should be pursued.

Notably, these considerations have limitations. First, we do not have data about a delay in treatment of fractures that do not reach a hospital soon after the trauma accident, and we did not collect data from other hospitals in our region that usually treat children from 12 to 18 years old, probably losing much of the data about fractures and treatments in this age range. We also did not consider weather patterns (March and April are usually lower-fracture-rate months compared to July or August), nor did we collect data about rainfalls and sunny days in 2018, 2019 and 2020. Lastly, we only considered rates of accidental trauma, which did not include any form of abuse, during the lockdown period.

## 5. Conclusions

In conclusion, home confinement decreased admissions to the emergency department for trauma by shutting down outdoor activities, schools and sports activities. The volume of paediatric patients seen for trauma during the lockdown decreased by about 75% from two years prior. Moreover, the patients who were seen during the lockdown were significantly younger children, probably because only children with major trauma were brought to the hospital by their parents to avoid exposure to the virus, whereas children with contusions and sprains were treated conservatively at home. This study finally demonstrates how a lockdown could differently affect children according to the age group.
